# Supplementary material for: HIVprotI: an integrated web based platform for prediction and design of HIV proteins inhibitors
Source: J Cheminform. 2018 Mar 9;10:12. doi: 10.1186/s13321-018-0266-y (PMC5845081; doi:10.1186/s13321-018-0266-y)
Supplement: Supplementary file 2 — Additional file 2. Source code of HIVProtI web server. [file 13321_2018_266_MOESM2_ESM.zip › HIVprotI_Source-code/hivprotI/weka-3-6-6/doc/weka/associations/tertius/SimpleLinkedList.html]

SimpleLinkedList


---


|  |  |  |
| --- | --- | --- |
| **Overview** \| **Package** \| **Class** \| **Tree** \| **Deprecated** \| **Index** \| **Help** \| **Weka's home** | |  |
| **PREV CLASS**   **NEXT CLASS** | **FRAMES**    **NO FRAMES**     **All Classes** |  |
| SUMMARY: NESTED \| FIELD \| CONSTR \| METHOD | DETAIL: FIELD \| CONSTR \| METHOD |  |


---


## weka.associations.tertius Class SimpleLinkedList

```
java.lang.Object
  weka.associations.tertius.SimpleLinkedList
```

**All Implemented Interfaces:**: java.io.Serializable, RevisionHandler

---

``` public class SimpleLinkedList extends java.lang.Object implements java.io.Serializable, RevisionHandler ```

**Version:**
:   $Revision: 1.6 $

**Author:**
:   Peter A. Flach, Nicolas Lachiche

**See Also:**: Serialized Form

---

| **Nested Class Summary** | |
| --- | --- |
| `class` | `SimpleLinkedList.LinkedListInverseIterator` |
| `class` | `SimpleLinkedList.LinkedListIterator` |


**Constructor Summary**

`SimpleLinkedList()`


| **Method Summary** | |
| --- | --- |
| `void` | `add(java.lang.Object o)` |
| `void` | `addAll(SimpleLinkedList list)` |
| `void` | `addFirst(java.lang.Object o)` |
| `void` | `clear()` |
| `java.lang.Object` | `getFirst()` |
| `java.lang.Object` | `getLast()` |
| `java.lang.String` | `getRevision()`             Returns the revision string. |
| `SimpleLinkedList.LinkedListInverseIterator` | `inverseIterator()` |
| `boolean` | `isEmpty()` |
| `SimpleLinkedList.LinkedListIterator` | `iterator()` |
| `void` | `merge(SimpleLinkedList list, java.util.Comparator comp)` |
| `java.lang.Object` | `removeFirst()` |
| `int` | `size()` |
| `void` | `sort(java.util.Comparator comp)` |
| `java.lang.String` | `toString()` |

**Methods inherited from class java.lang.Object**

`equals, getClass, hashCode, notify, notifyAll, wait, wait, wait`

**Constructor Detail**

### SimpleLinkedList

```
public SimpleLinkedList()
```


**Method Detail**

### removeFirst

```
public java.lang.Object removeFirst()
```

---


### getFirst

```
public java.lang.Object getFirst()
```

---


### getLast

```
public java.lang.Object getLast()
```

---


### addFirst

```
public void addFirst(java.lang.Object o)
```

---


### add

```
public void add(java.lang.Object o)
```

---


### addAll

```
public void addAll(SimpleLinkedList list)
```

---


### clear

```
public void clear()
```

---


### isEmpty

```
public boolean isEmpty()
```

---


### iterator

```
public SimpleLinkedList.LinkedListIterator iterator()
```

---


### inverseIterator

```
public SimpleLinkedList.LinkedListInverseIterator inverseIterator()
```

---


### size

```
public int size()
```

---


### merge

```
public void merge(SimpleLinkedList list,
                  java.util.Comparator comp)
```

---


### sort

```
public void sort(java.util.Comparator comp)
```

---


### toString

```
public java.lang.String toString()
```

:   **Overrides:**: `toString` in class `java.lang.Object`

---


### getRevision

```
public java.lang.String getRevision()
```

:   Returns the revision string.

    :   **Specified by:**: `getRevision` in interface `RevisionHandler`
    :   **Returns:**: the revision


---


|  |  |  |
| --- | --- | --- |
| **Overview** \| **Package** \| **Class** \| **Tree** \| **Deprecated** \| **Index** \| **Help** \| **Weka's home** | |  |
| **PREV CLASS**   **NEXT CLASS** | **FRAMES**    **NO FRAMES**     **All Classes** |  |
| SUMMARY: NESTED \| FIELD \| CONSTR \| METHOD | DETAIL: FIELD \| CONSTR \| METHOD |  |


---
